# A Novel Small Compound SH-2251 Suppresses Th2 Cell-Dependent Airway Inflammation through Selective Modulation of Chromatin Status at the *Il5* Gene Locus

**DOI:** 10.1371/journal.pone.0061785

**Published:** 2013-04-16

**Authors:** Junpei Suzuki, Makoto Kuwahara, Soichi Tofukuji, Masashi Imamura, Fuminori Kato, Toshinori Nakayama, Osamu Ohara, Masakatsu Yamashita

**Affiliations:** 1 Department of Pharmacogenomics, Graduate School of Pharmaceutical Science, Chiba University, Chuo-ku, Chiba, Japan; 2 Department of Human Genome Research, Kazusa DNA Research Institute, Kisarazu, Chiba, Japan; 3 Department of Immunology, Graduate School of Medicine, Chiba University, Chuo-ku, Chiba, Japan; 4 Central Research Institute, Ishihara Sangyo Kaisha, Ltd., Kusatsu, Shiga, Japan; 5 CREST, Japan Science and Technology Agency, Chuo-ku, Chiba, Japan; 6 Department of Immunology, Graduate School of Medicine, Ehime University, Toon, Ehime, Japan; 7 PRESTO, Japan Science and Technology Agency, Toon, Ehime, Japan; Fundação Oswaldo Cruz, Brazil

## Abstract

IL-5 is a key cytokine that plays an important role in the development of pathological conditions in allergic inflammation. Identifying strategies to inhibit IL-5 production is important in order to establish new therapies for treating allergic inflammation. We found that SH-2251, a novel thioamide-related small compound, selectively inhibits the differentiation of IL-5-producing Th2 cells. SH-2251 inhibited the induction of active histone marks at the *Il5* gene locus during Th2 cell differentiation. The recruitment of RNA polymerase II, and following expression of the Th2 cell-specific intergenic transcripts around the *Il5* gene locus was also inhibited. Furthermore, Th2 cell-dependent airway inflammation in mice was suppressed by the oral administration of SH-2251. Gfi1, a transcriptional repressor, was identified as a downstream target molecule of SH-2251 using a DNA microarray analysis. The Gfi1 expression dramatically decreased in SH-2251-treated Th2 cells, and the SH-2251-mediated inhibition of IL-5-producing Th2 cell differentiation was restored by transduction of *Gfi1*. Therefore, our study unearthed SH-2251 as a novel therapeutic candidate for allergic inflammation that selectively inhibits active histone marks at the *Il5* gene locus.

## Introduction

Asthma is a complex chronic inflammatory disease characterized by airway inflammation and hyperresponsiveness obstruction that affects approximately 300 million individuals worldwide [1]. A large number of clinical studies and animal experimental models support a central role of antigen-specific Th2 cells in the pathological responses of atopic asthma [2], [3]. In particular, antigen-specific effector and memory Th2 cells appear to play an important role in initiating allergic inflammatory status in the early stage of atopic asthma. Although eliminating Th2 cells and/or inhibiting Th2 cell functions at the early stage of atopic asthma may lead to complete remission, strategies for modulating Th2 cell numbers and/or functions have not been established.

IL-5 is a hematopoietic cytokine that exerts important effects on eosinophils and basophils. IL-5 induces differentiation and maturation of eosinophils in bone marrow, migration to tissue sites and prevention of eosinophil apoptosis [4] [5]. IL-5 also plays a role in the development, metabolism, and function of basophils [6]. Eosinophilic inflammation is a hallmark of asthma that correlates with bronchial hyperresponsiveness and disease severity. In an asthma model, IL-5-deficient mice did not display eosinophilia, airway hyperreactivity or pulmonary injury, in contrast to that observed in control mice [7]. Treatment of mice with anti-IL-5 mAb also results in decreases in eosinophilic inflammation that are associated with reduced reactivity of methacholine. Therefore, IL-5 is a therapeutic target for allergic inflammation as well as hypereosinophilic syndrome.

Th2 cells produce IL-4, IL-5 and IL-13, and have been shown to play a crucial role in IgE production and eosinophil recruitment. Th2 cells are involved in clearance of extracellular parasites and also promote pathogenic responses associated with allergic inflammation. In peripheral CD4 T cells, IL-4-mediated activation of the transcription factor STAT6 induces the expression of *Gata3* mRNA, which drives Th2 cell differentiation [8]. GATA-3 binds to various regulatory regions on the Th2 cytokine gene loci and induces chromatin remodeling [9], [10], [11]. In addition, GATA-3 binds to the *Il5* promoter and acts as a transcriptional factor for IL-5 [12].

In addition to Th2 cells, a large number of cell types produce IL-5, including eosinophils [5] [4], natural killer (NK)T cells [13], nuocytes [14], natural helper (NH) cells [15] and IL-5-producing innate cells [16]. Recently, the IL-33-induced production of IL-5 from innate cells was reported. IL-33-mediated production of IL-5 plays critical roles in lung eosinophil regulation [16], lung inflammation [17] and protease allergen-induced airway inflammation [18]. In addition, the IL-33/IL-5 signaling pathway plays a crucial role in the disease pathogenesis of severe asthma that is resistant to high doses of inhaled corticosteroids but responsive to systemic corticosteroids and anti-IL-5 therapy [19].

Gfi1 is a DNA binding transcriptional repressor that plays important roles in several hematopoietic cells [20]. Gfi1 exerts its role as a transcriptional repressor by interacting with a number of histone modification enzyme including LSD-1/CoRest, G9a and HDACs [21], [22], [23]. It is well established that Gfi1 regulates the development of Th cell subsets. Zu et al. demonstrated that Gfi1 regulates Th2 cell expansion via enhancement of Stat5 activity [24]. However, the forced expression of constitutively active Stat5 fails to restore Th2 cell development in *Gfi1*-dificient CD4 T cells, possibly because Gfi1 might also play additional roles in Th2 cell development that are independent of Stat5. We previously reported that the expression level of Gata3 proteins and generation of IL-5-producing Th2 cells are severely impaired in *Gfi1*-deficient CD4 T cells [25]. The transduction of Gata3 into *Gfi1*-deficient Th2 cells partially restores the development of IL-5-producing Th2 cells, thus indicating that Gfi1 controls IL-5-producing Th2 cell generation in part through regulation of the Gata3 protein expression.

SH-2251, a thioamide-related compound, was originally synthesized as an inhibitor of IL-5 production. However, the molecular mechanisms by which SH-2251 inhibits IL-5 production and the effects of SH-2251 on Th2 cell differentiation remain to be elucidated. We herein investigated the effects of SH-2251 on Th2 cell differentiation and demonstrated that SH-2251 negatively regulates IL-5-producing Th2 cell differentiation and chromatin remodeling at the *Il5* gene locus. Furthermore, we demonstrated that Th2 cell-dependent allergic airway inflammation is suppressed by oral administration of SH-2251. A DNA microarray analysis revealed that SH-2251 inhibits the differentiation of IL-5-producing Th2 cells via repression of the Gfi1 expression. Therefore, SH-2251 belongs to a unique class of inhibitors of Th2-dependent immune responses that modulate chromatin remodeling at the *Il5* gene locus and the subsequent the differentiation of IL-5 producing Th2 cells.

## Results

### SH-2251 selectively inhibits the generation of IL-5-producing Th2 cells

**Figure 1:**
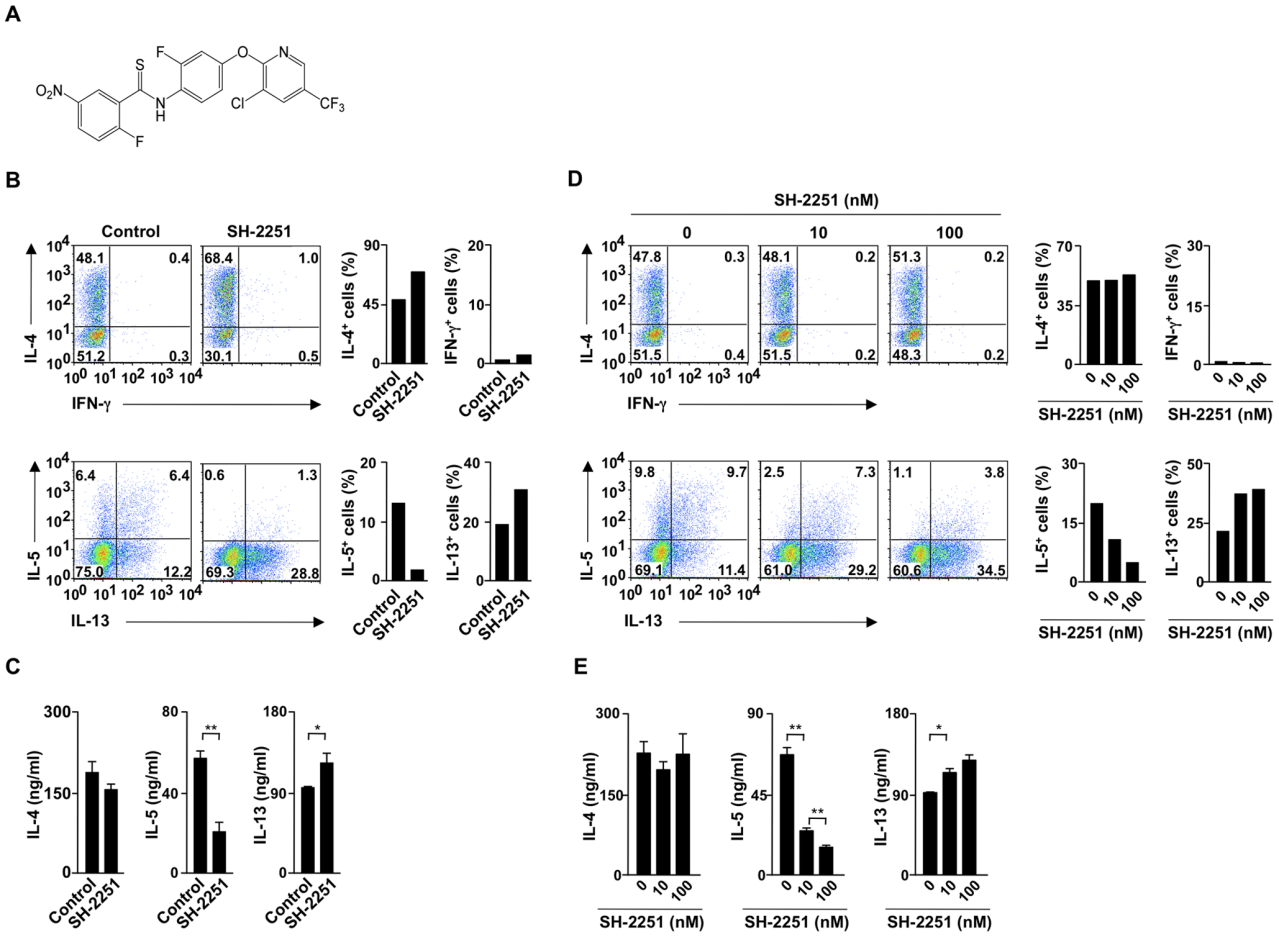
SH-2251 inhibits the generation of IL-5-producing Th2 cells. **(A)**, The chemical structure of SH-2251. **(B)**, Naïve CD4 T cells were cultured under Th2-conditions in the presence or absence of SH-2251 (100 nM) for five days. The cells were restimulated with an immobilized anti-TCR-β mAb for six hours, and the intracellular staining profiles of IL-4/IFN-γ (upper panel) and IL-5/IL-13 (lower panel) were determined using intracellular staining, respectively. The percentages of each quadrant are indicated. The average percentages of the generated cytokine-producing cells of three independent experiments are also shown with the standard deviation (right). **(C)**, Cytokine production induced by the SH-2251-treated Th2 cells shown in panel (B) was determined with ELISA. **(D)**, Naïve CD4 T cells were cultured under Th2-conditions in the presence of the indicated concentrations of SH-2251 for five days. The intracellular profiles were determined as described (B). The average percentage of three independent experiments of the generated cytokine-producing cells are also shown with the standard deviation **(E)**, Cytokine production by the SH-2251-treated Th2 cells shown in panel (D) was determined with ELISA. *P<0.05 and **P<0.01 (Student's *t*-test). Three independent experiments (C and E) were performed with similar results.

SH-2251 (**Fig. 1A**), a novel thioamide-related compound, was originally synthesized as an inhibitor of IL-5 production. However, the effects of SH-2251 on Th2 cell differentiation were not determined. To assess the effects of SH-2251 on Th2 cell differentiation, naïve CD4 T cells were purified and cultured under Th2-conditions in the presence or absence of SH-2251 for five days, and the ability to produce Th2 cytokines was determined using intracellular staining. As shown in **Fig. 1B**, the generation of IL-5-producing Th2 cells decreased in the SH-2251-treated cultures, whereas the number of IL-4- and IL-13-producing cells slightly increased. The selective reduction of IL-5 production was also confirmed on ELISA (**Fig. 1C**). The generation of IFN-γ-producing Th1 cells and IL-17A-producing Th17 cells was moderately decreased, while development of IL-9-producing Th9 cells was augmented by treatment with SH-2251 (**Fig. S1A–C in File S1**). To determine the optimal concentration for inhibition of IL-5-producing Th2 cell differentiation, naïve CD4 T cells were cultured under Th2-conditions in the presence of the indicated concentrations of SH-2251. Inhibitory effects were observed at the 10 nM concentration of SH-2251 and peaked at 100 nM (**Fig. 1D**). Dose-dependent effects of SH-2251 on the inhibition of IL-5 induction were also confirmed using ELISA (**Fig. 1E**). The production of IL-4 and IL-13 was not impaired (**Fig. 1E**). These results indicate that SH-2251 inhibits IL-5-producing Th2 cell differentiation without inhibiting the generation of IL-4- or IL-13-producing Th2 cells.

### SH-2251 selectively inhibits induction of active histone modifications at the *Il5* gene locus during Th2 cell differentiation

**Figure 2:**
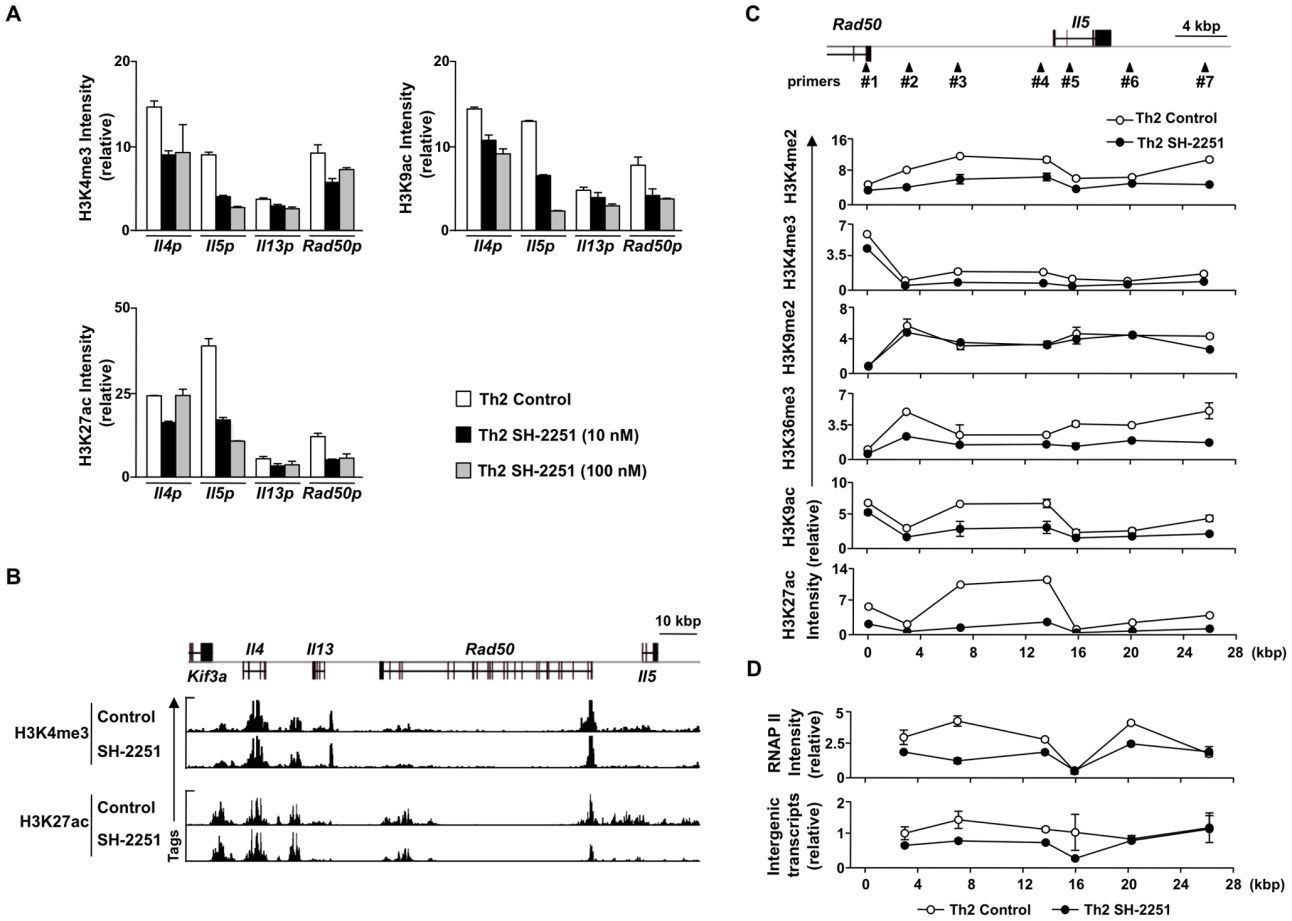
The induction of active histone marks at the *Il5* gene locus is inhibited by SH-2251. **(A)**, Naïve CD4 T cells were cultured under Th2-conditions for five days in the presence of the indicated concentration of SH-2251, and a ChIP assay was performed with the indicated antibodies. The relative intensity (/Input) is shown with the standard deviation. **(B)**, The global patterns of histones H3K4me3 and H3K27ac at the Th2 cytokine gene loci were determined using ChIP-sequencing. **(C)**, The indicated histone modification status around the *Il5* gene locus in the SH-2251-treated Th2 cells was determined using a manual ChIP assay. The relative intensity (/Input) is shown with the standard deviation. **(D)**, Recruitment of RNA polymerase II around the *Il5* gene locus (upper panel) was determined using a manual ChIP assay. The relative intensity (/Input) is shown with the standard deviation. The transcripts around the *Il5* gene in the SH-2251-treated Th2 cells (lower panel) were determined using quantitative RT-PCR. The relative intensity (/*Hprt*) is shown with the standard deviation. Four independent experiments (A, C and D) were performed with similar results.

Changes in histone modification are a marker of chromatin remodeling [26], [27]. During Th2 cell differentiation, active histone modifications including histones H3K4me2/3, H3K9ac and H3K27ac, are induced at Th2 cytokine gene loci [9] [11]. We examined the effect of SH-2251 on the induction of active histone modifications during Th2 cell differentiation. As shown in **Fig. 2A**, the levels of active histone modifications such as those of H3K4me3, H3K9ac and H3K27ac at the *Il5* promoter were reduced by treatment with SH-2251 in a dose-dependent manner. The levels of H3K9ac and H3K27ac, but not H3K4me3, at the Rad50 promoter decreased (**Fig. 2A**). In sharp contrast, the active histone modifications at the *Il4* and *Il13* promoters were unaffected by SH-2251 treatment (**Fig. 2A**). To confirm the selective effects of SH-2251 on the levels of active histone modifications around the *Il5* gene locus, we performed ChIP-sequencing with anti-histone H3K4me3 pAb and H3K27ac pAb. Decreased levels of H3K4me3 and H3K27ac were detected from the 5′ region of the *Rad50* gene to the *Il5* gene, while reduced levels were spread over the down stream region of the *Il5* gene locus in the SH2251-treated Th2 cells (**Fig. 2B** **and Fig. S2 in File S1**). Reduction in the levels of H3K4me3 and H3K27ac around the *Il5* gene locus in the SH-2251-treated Th2 cells were confirmed using a manual ChIP analysis (**Fig. 2C**). Changes in other histone modifications, including H3K4me2, H3K9me2, H3K36me3 and H3K9ac, around the *Il5* gene locus were also determined with a manual ChIP analysis. The levels of active histone marks such as those of H3K4me2, H3K36me3 and H3K9ac around the *Il5* gene locus were decreased in the SH-2251-treated Th2 cells (**Fig. 2C**). The level of H3K9me2 was not affected by treatment with SH-2251 (**Fig. 2C**). No obvious signals were detected with an anti-H3K27me3 pAb (data not shown). Finally, we assessed the effects of SH-2251 treatment on the recruitment of RNA polymerase II (PolII) and subsequent intergenic transcription around the *Il5* gene locus. SH-2251 reduced the recruitment of polII (**Fig. 2D** **upper panel**) and the level of transcription (**Fig. 2D** **lower panel**) in the Th2 cells. These results suggest that SH-2251 blocks the generation of IL-5-producing Th2 cells, presumably by inhibiting chromatin remodeling at the *Il5* gene locus.

### Th2-dependent airway inflammation is attenuated by the administration of SH-2251

**Figure 3:**
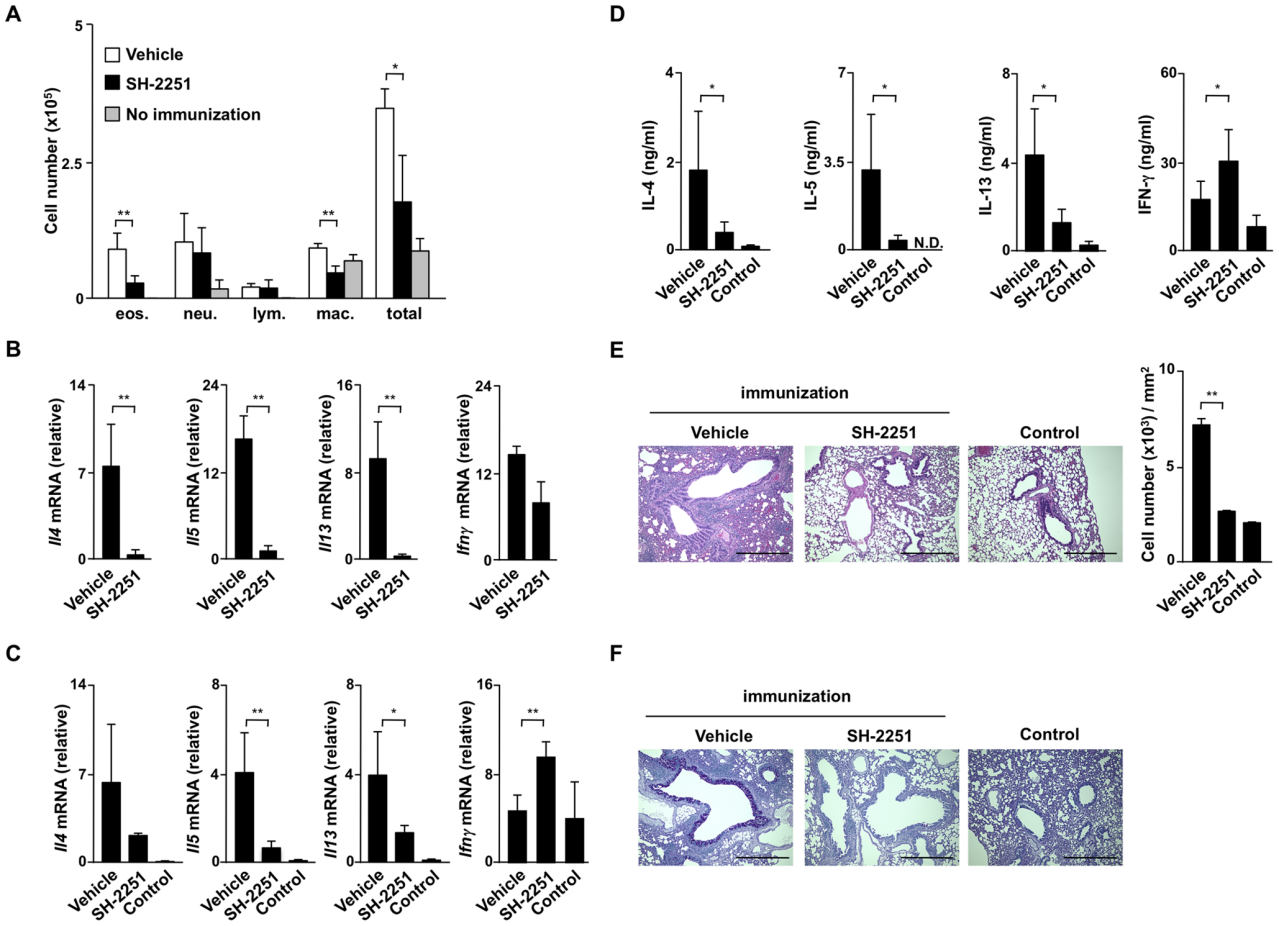
OVA-induced airway inflammation is attenuated by oral administration of SH-2251. **(A)**, Decreased infiltration of eosinophils in the BAL fluid of asthmatic SH-2251-administered mice. The absolute numbers of eosinophils (Eos.), neutrophils (Neu.), lymphocytes (Lym.) and macrophages (Mac.) in the BAL fluid are shown with standard deviations (n = 5 per group). *P<0.01 and **P<0.001 by ANOVA and the Bonferroni-test. (B), Quantitative RT-PCR of *Il4*, *Il5* and *Il13* mRNA in the BAL fluid cells of vehicle and SH-2251-administered mice. (C), Quantitative RT-PCR of *Il4*, *Il5*, *Il13* and *Ifnγ* mRNA in the lung CD4 T cells of vehicle and SH-2251-administered mice. (n = 5 per group). (D), Cytokine production from lung CD4 T cells of vehicle and SH-2251-administered mice stimulated *in vitro*. The lung CD4 T cells were stimulated with immobilized anti-TCR-β mAb for 48 hours and the concentrations of cytokines in the culture supernatants were determined using ELISA. The lungs were fixed and stained with hematoxylin and eosin (E, left) or periodic acid-Schiff reagent (F). The scale bars represent 500 µm. The numbers of infiltrated leukocytes in the peribronchiolar regions are shown (mean cell numbers/mm^2^) (E, right). Three independent experiments were performed with similar results. Student's *t*-test was used for the statistical analyses. *P<0.05 and **P<0.01 (B, C, D and E)

We next investigated the effects of the oral administration of SH-2251 (10 mg/kg) in mice model of airway inflammation. BALB/c mice were immunized with OVA absorbed by alum, then challenged with OVA intranasally. We observed decreases in the infiltration of inflammatory cells, including eosinophils, in the bronchoalveolar lavage (BAL) fluid of the OVA-immunized SH-2251-treated mice in comparison to that observed in the vehicle-administrated control group (**Fig. 3A**). The expressions of *Il4*, *Il5* and *Il13* mRNA in the BAL fluid cells were also very low, whereas the reduction of *Ifnγ* was marginal in the SH-2251-administered group (**Fig. 3B**). A reduced expression of *eosinophil peroxidase* (*Epo*) mRNA in the BAL fluid cells of the SH-2251-administered mice supported decreased infiltration of eosinophils (**Fig. S3A in File S1**). We prepared CD4 T cells from the lungs of OVA-challenged mice to confirm the effects of SH-2251 administration. The expressions of mRNA for Th2 cytokines in the CD4 T cells purified from the lung tissue were reduced in the SH-2251-treated mice (**Fig. 3C**). The purified CD4 T cells were further stimulated with immobilized anti-TCR-β mAb for 48 hours *in vitro*, and the production of cytokines was determined using ELISA. The level of IL-5 production was low in the CD4 T cells obtained from the SH-2251-administered mice in comparison to that observed in the CD4 T cells obtained from the vehicle-treated mice (**Fig. 3D**). In addition, the productions of IL-4 and IL-13 were also significantly decreased in the lung CD4 T cells obtained from the SH-2251-treated mice, whereas the IFN-γ production was increased (**Fig. 3D**). The number of mononuclear cells infiltrating the peribronchiolar regions of the lungs was reduced by SH-2251 administration (**Fig. 3E**). Both mucus hyper-production and goblet cell metaplasia, as assessed with PAS staining, were lower in the bronchioles of the SH-2251-administrered mice compared to that observed in bronchioles of the vehicle-treated control mice (**Fig. 3F**). The serum levels of anti-OVA immunoglobulin were unaffected by the administration of SH-2251 (**Fig. S3B in File S1**). These results indicate that the oral administration of SH-2251 can suppress Th2 cell-mediated allergic airway inflammation.

### The expression and functions of Gata3 are not influenced by SH-2251 treatment

**Figure 4:**
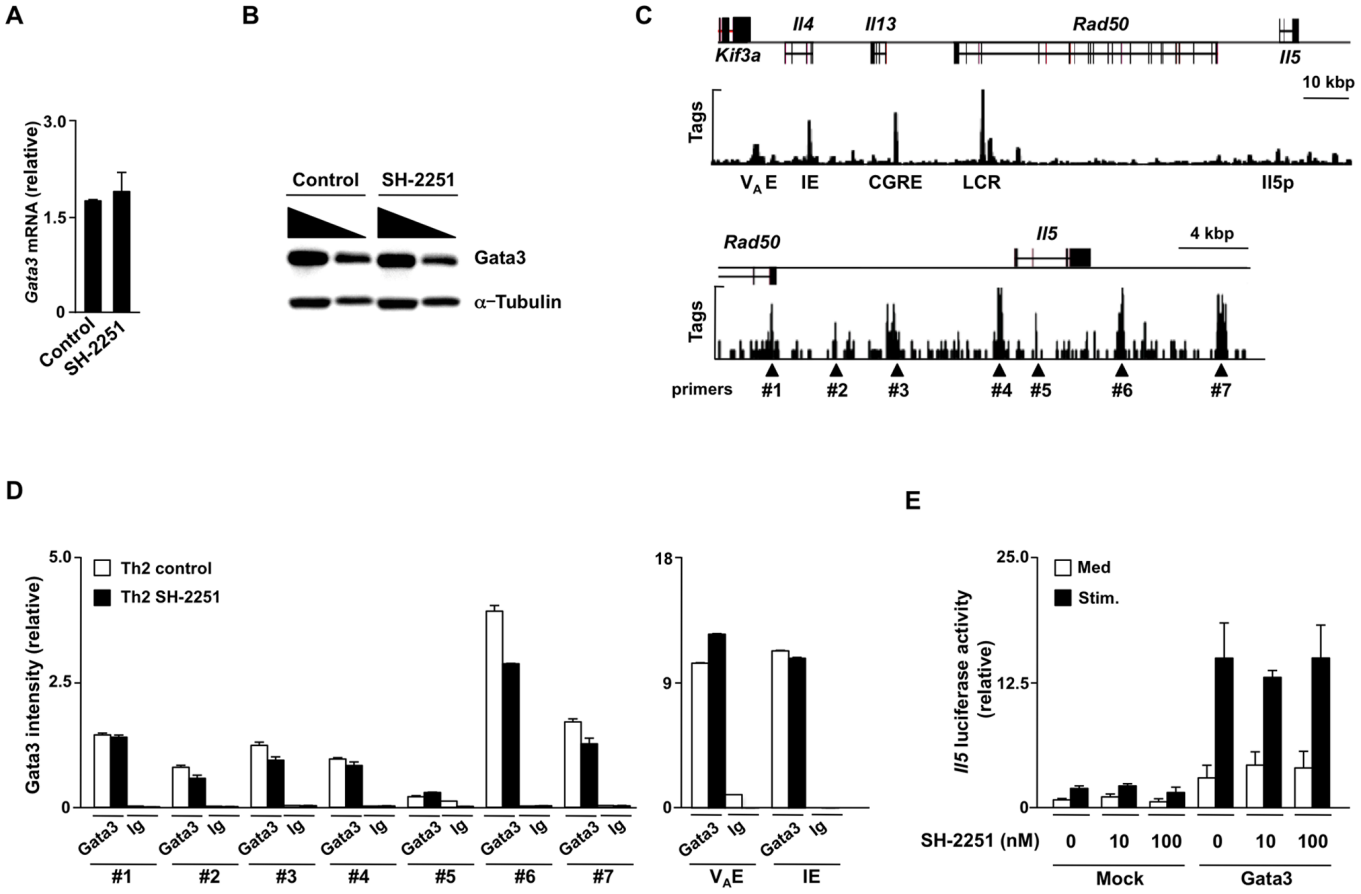
The expression and functions of Gata3 are not impaired by treatment with SH-2251. **(A)**, The mRNA expression of Gata3 in the SH-2251-trearted Th2 cells was determined using quantitative RT-PCR. The relative intensity (/*Hprt*) is shown with the standard deviation. **(B)**, The protein expression level of Gata3 was determined with immunoblotting. The nuclear (Gata3) and cytoplasmic (α-Tubulin) lysates with a three fold serial dilution were used. Three independent experiments (A and B) were performed with similar results. **(C)**, The global patterns of Gata3 binding at the Th2 cytokine gene loci (upper panel) and the *Il5* gene locus (lower panel) were determined using ChIP-sequencing with an anti-Gata3 pAb. The locations of PCR primer pairs (triangle) used in a manual ChIP assay are also listed. **(D)**, The binding of Gata3 around the *Il5* gene locus (left) panel and the V_A_ enhancer (V_A_ E) and intronic enhancer (IE) regions of the *Il4* gene locus (right panel) in the SH-2251-treated Th2 cells was determined using a manual ChIP assay. The relative intensity (/Input) is shown with the standard deviation. Three independent experiments were performed with similar results. **(E)**, The effects of SH-2251 on the Gata3-dependent transcriptional activation of the *Il5* promoter were determined using a Dual luciferase assay. The mean and standard deviation of the relative luciferase activity of three different experiments are shown. Stim: PMA (30 ng/ml)+dbcAMP (100 µM). Four independent experiments (A, B, D and E) were performed with similar results.

Gata3 plays an essential role in the induction of chromatin remodeling at the Th2 cytokine gene locus following Th2 cell differentiation [9] [10] [11]. In addition, Gata3 induces the transcriptional activation of the *Il5* gene [12]. In this study, treatment with SH-2251 showed no effects on the Gata3 mRNA (**Fig. 4A**) or protein (**Fig. 4B**) expressions in the Th2 cells. Next, we wanted to determine the effects of SH-2251 on binding of Gata3 at the Th2 cytokine gene locus. The binding of Gata3 throughout the Th2 cytokine gene locus was determined comprehensively using ChIP-sequencing with an anti-Gata3 pAb. Gata3 has been reported to bind to the V_A_ enhancer [28], *Il4* intron2 [29], CGRE [30], Th2 LCR [31], [32] and *Il5* promoter regions [33] in Th2 cells. The binding of Gata3 at these regions was confirmed with ChIP-sequencing (**Fig. 4C** **upper panel**). In addition, we newly identified several Gata3 binding genomic regions around the *Il5* gene locus (**Fig 4C** **lower panel**: #1∼#7). The binding of Gata3 at these regions in the Th2 cells was not inhibited by SH-2251 treatment (**Fig. 4D**). Finally, we examined whether SH-2251 can inhibit the Gata3-induced transcriptional activation of the *Il5* promoter using a reporter gene analysis. As indicated in **Fig. 4E**, SH-2251 showed only marginal effects on the Gata3-dependent activation of the *Il5* promoter. These data suggest that Gata3 is unlikely to be a target of SH-2251 in the inhibition of IL-5-producing Th2 cell development.

### A decreased expression of Gfi1 is involved in the SH-2251-mediated inhibition of IL-5-producing Th2 cell generation

**Figure 5:**
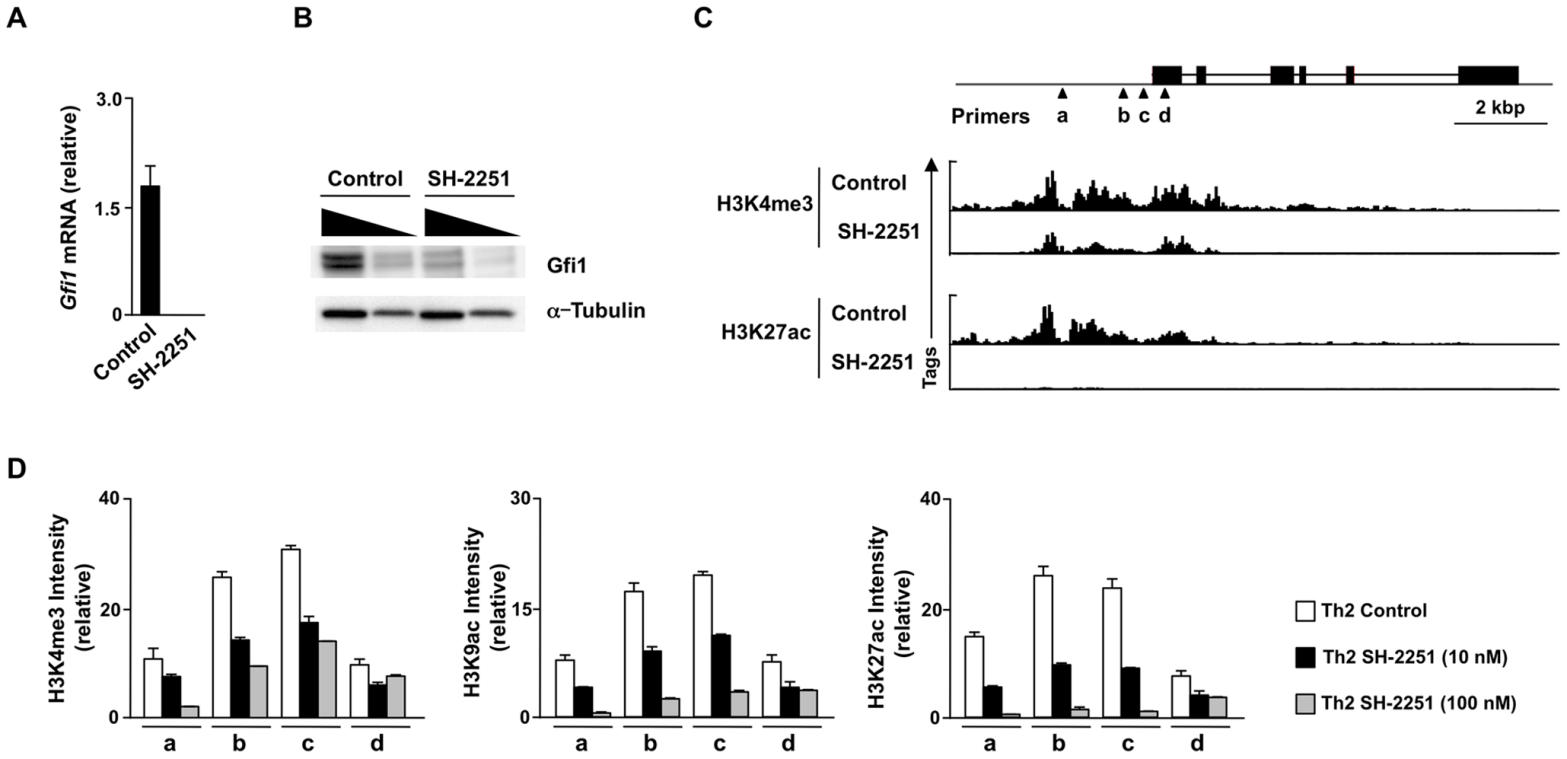
Gfi1 is a downstream target of SH-2251. **(A)**, A decreased *Gfi1* mRNA expression in the SH-2251 treated Th2 cells. The expression of *Gfi1* was determined with quantitative RT-PCR. The relative intensity (/*Hprt*) is shown with the standard deviation. **(B)**, A decreased level of Gfi1 proteins was detected with immunoblotting. The nuclear (Gfi1) and cytoplasmic (α-Tubulin) lysates with a three fold serial dilution were used. **(C)**, The global patterns of the histone H3K4me3 and H3K27ac levels at the *Gfi1* gene locus were determined using ChIP-sequencing. The locations of PCR primer pairs (triangle) used in a manual ChIP assay are also listed. **(D)**, Manual ChIP assays were performed with anti-histone H3K4me3, H3K9ac or H3K27ac pAb. The relative intensity (/Input) is shown with the standard deviation. Four independent experiments (A, B and D) were performed with similar results.

We conducted a DNA microarray analysis to identify the target gene(s) that are involved in the SH-2251-mediated inhibition of IL-5-producing Th2 cell generation. We found that the expression of *Gfi1* mRNA was dramatically decreased in the SH-2251-treated Th2 cells (**Fig. 5A**). A reduction in the Gfi1 protein expression was also induced by SH-2251-treatment (**Fig. 5B**). To assess the molecular mechanisms by which SH-2251 inhibits the *Gfi1* expression, the histone modifications present at the *Gfi1* gene locus were determined using ChIP-sequencing with an anti-histone H3K27ac pAb or an H3K4me3 pAb, respectively. As shown in **Fig. 5C** **lower panel**, a striking reduction in the histone H3K27ac level at the *Gfi1* gene locus in the SH-2251-treated Th2 cells was detected. The level of H3K4me3 at the *Gfi1* gene locus was moderately decreased (**Fig. 5C** **upper panel**). A dose-dependent inhibition of the H3K4me3 and H3K27ac levels at the *Gfi1* gene locus induced by SH-2251 was confirmed using a manual ChIP assay (**Fig. 5D**). The level of H3K9ac at the *Gfi1* gene locus was also inhibited by SH-2251 treatment (**Fig. 5D**). As indicated in **Fig. S4 in File S1**, the levels of histone H3K27ac and H3K4me3 at the *Gfi1* gene locus were higher in the Th2 cells than that in the naïve CD4 T cells. The levels of histone H3K27ac and H3K4me3 modifications in the SH-2251-treated Th2 cells were almost comparable to those in the naïve CD4 T cells (**Fig. S4 in File S1**), thus indicating that SH-2251 inhibits the induction of the histone H3K27ac at the *Gfi1* locus during Th2 cell differentiation.

### Transduction of *Gfi1* into SH-2251-treated Th2 cells restores IL-5 production

**Figure 6:**
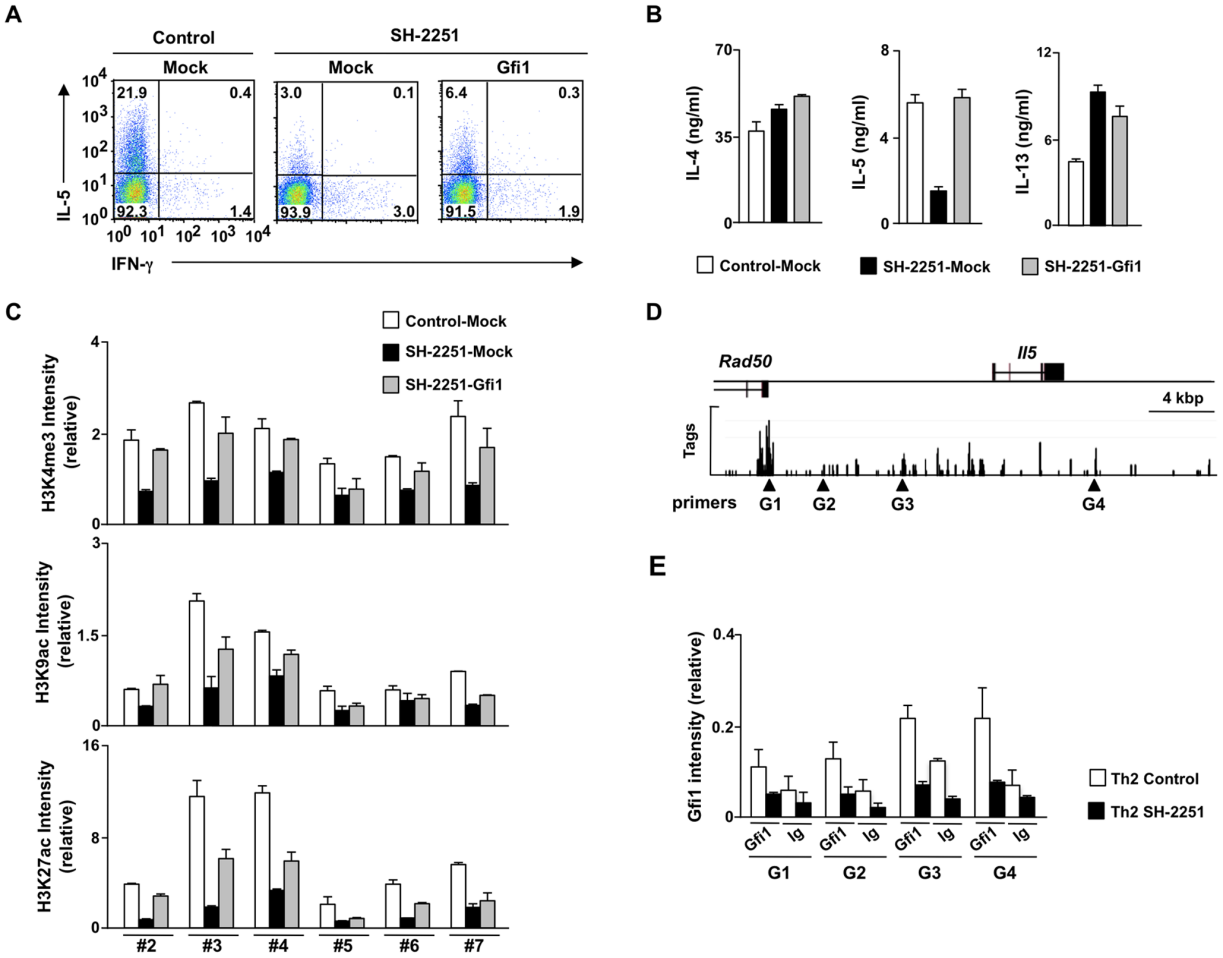
Transduction of *Gfi1* into SH-2251-treated Th2 cells restores the differentiation of IL-5-producing Th2 cells. **(A)**, CD4 T cells were cultured under Th2-conditions in the presence or absence of SH-2251 (100 nM) for two days, then the cells were transduced with Mock- or *Gfi1*-IRES-hNGFR-containing retrovirus vectors. Three days after transduction, the IL-5/IFN-γ staining profiles of the transduced cells (hNGFR-positive cells) were determined with intracellular staining. The percentages of cells in each quadrant are indicated. **(B)**, The cytokine production from SH-2251-treated Th2 cells transduced with *Gfi1* was determined. **(C)**, Histones H3K4me3, H3K9ac and H3K27ac at the *Il5* gene locus in hNGFR-positive *Gfi1*-transduced SH-2251-treated Th2 cells. The relative intensity (/Input) is shown with the standard deviation. Three (A, B and C) independent experiments were performed with similar results. **(D)**, The global pattern of Gfi1 binding around the *Il5* gene locus was determined using ChIP-sequencing with an anti-Gfi1 mAb. The locations of the PCR primer pairs (triangle) used in a manual ChIP assay are also listed. **(E)**, The binding of Gfi1 around the *Il5* gene locus in SH-2251-treated Th2 cells was determined using a manual ChIP assay. The relative intensity (/Input) is shown with the standard deviation. Two independent experiments were performed with similar results.

To elucidate the role of Gfi1 reduction in the SH-2251-mediated inhibition of IL-5-producing Th2 cell differentiation, we transduced *Gfi1* into SH-2251-treated Th2 cells using retrovirus vectors and measured the IL-5 production ability. As shown in **Fig. 6A**, the transduction of *Gfi1* into the SH-2251-treated Th2 cells partially restored the generation of IL-5 producing Th2 cells. The production levels of IL-5 in the SH-2251-treated Th2 cells were completely restored by the transduction of *Gfi1* (**Fig. 6B**). The production of IL-4 and IL-13 in the *Gfi1*-transduced Th2 cells was not altered in comparison to that observed in the *Mock*-transduced SH-2251-treated Th2 cells (**Fig. 6B**). The levels of histone H3K4me3 and H3K27ac around the *Il5* gene locus were also ameliorated in the *Gfi1*-transduced SH-2251-treated Th2 cells (**Fig. 6C**). Histones H3K4me3, H3K9ac and H3K27ac at the *Il4* and *Il13* promoters were not influenced by the transduction of Gfi1 (**Fig. S5 in File S1**). To examine the molecular mechanisms by which Gfi1 controls the histone modification status at the *Il5* gene locus, the binding of Gfi1 around the *Il5* gene locus was determined using a ChIP-sequence analysis with an anti-Gfi1 pAb. Low, but reproducible binding of Gfi1 was detected around the *Il5* gene locus in the Th2 cells (**Fig. 6D**). The binding of Gfi1 around the *Il5* gene locus in the Th2 cells decreased by the treatment with SH-2251 (**Fig. 6E**). These results suggest that SH-2251 inhibits the chromatin remodeling at the *Il5* gene locus and subsequent IL-5-producing Th2 cell differentiation in part by attenuating the Gfi1 expression.

## Discussion

We herein demonstrated that a thioamide-related small chemical compound, SH-2251, inhibits the differentiation of IL-5-producing Th2 cells by attenuating the Gfi1 expression. Treatment of developing Th2 cells with SH-2251 reduced the generation of IL-5-producing Th2 cells and the expression of Gfi1. SH-2251 also inhibited induction of active histone modifications at the *Il5* gene locus as well as the *Gfi1* locus in developing Th2 cells. We found that Gfi1 binds to several genomic regions around the *Il5* gene locus in Th2 cells, which was reduced by treatment with SH-2251. We previously reported that the induction of histones H3K4me3 and H3K9/14ac at the *Il5* gene locus and subsequent IL-5-producing Th2 cell differentiation are impaired in *Gfi1*-deficient CD4 T cells [25]. In addition, in this study, retrovirus vector-mediated transduction of *Gfi1* into SH-2251-treated developing Th2 cells restored the levels of active histone modifications at the *Il5* gene locus, and subsequent generation of IL-5-producing Th2 cells. Therefore, the *Gfi1*-*Il5* axis is a target for SH2251-mediated inhibition of IL-5-producing Th2 cell differentiation.

Clinical trials of the anti-IL-5 mAb have demonstrated therapeutic benefits across a spectrum of eosinophil-related disorders [34]. Recently, a result of a clinical trial of a humanized anti-IL-5 mAb (Mepolizumab) was reported [35]. Although no changes in airway hyperresponsiveness were noted, reductions in blood/sputum eosinophilia and the number of asthma exacerbations occurring during the year were reported. In addition, reductions in airway wall thickness were also observed. These results indicate that the neutralization of IL-5 might have a positive impact on airway remodeling. We demonstrated the inhibitory effects of SH-2251 on the generation of IL-5-producing Th2 cells and IL-5 production. Furthermore, the oral administration of SH-2251 was found to suppress OVA-induced allergic airway inflammation in a mice model. These data suggest that SH-2251 is a novel therapeutic candidate for diseases involving allergic inflammation, including asthma.

The generation of IL-13-producing cells and IL-13 production were augmented by SH-2251 treatment *in vitro*. The IL-13 production moderately increased in the *Gfi1*-deficient CD4 T cells (M.Y. unpublished observation), suggesting that Gfi1 may inhibit IL-13 production in CD4 T cells. However, our *in vivo* experimental results demonstrated a reduction in IL-13 production induced by the administration of SH-2251. In addition, the production of IL-4 was also moderately decreased *in vivo*. A DNA microarray analysis indicated a reduced expression of *Ccr3* mRNA in the SH-2251-treated Th2 cells. Therefore, it is likely that SH-2251 exerts some effects on the expressions of chemokine receptors in Th2 cells and that recruitment of Th2 cells to the inflamed sites is inhibited. In addition, it is possible that SH-2251 also affects the function of antigen-presenting cells. Taken together, although an SH-2251-mediated increase in IL-13 production was detected in the *in vitro* experiments, the administration of SH-2251 provides beneficial effects in the treatment of asthmatic patients.

Lung epithelial cells can produce multiple cytokines, including IL-25 and IL-33, in response to various stressors. The intranasal administration of IL-25 induces asthmatic symptoms [36], and anti-IL-25 antibody treatment suppresses OVA-induced allergic inflammation [37]. It is thought that IL-25 acts on NKT cells and promotes Th2 cytokine production [38]. Recently, the IL-33-mediated production of IL-5 has been reported to play a critical role in lung eosinophil regulation [16], lung inflammation [17] and protease allergen-induced airway inflammation [18]. Gfi1, a downstream target of SH-2251, is broadly expressed in hematopoietic lineage cells, and *Gfi1* knockout animals display many abnormalities, including neutropenia, T cell development defects, hematopoietic stem cell defects and defects in dendritic cell development and functions [20]. It is likely that Gfi1 is also expressed in NKT cells, NH cells, neuocytes and IL-5-producing innate cells. Therefore, it is interesting to examine whether the treatment of SH-2251 can inhibit both the IL-25- and IL-33-induced production from these cell populations.

SH-2251 inhibits the generation of IL-5-producing Th2 cells, in part by repressing Gfi1 induction. Gfi1 is induced by the TCR-mediated activation of the ERK MAPK cascade [25]. In this study, although SH-2251 inhibited the Gfi1 expression, the inhibitory activity for ERK MAPK was very weak (IC_50_>1 µM; M.I. and F.K. personal communication). The activation of the Ras-ERK MAPK cascade also prevents the ubiquitin/proteasome-dependent degradation of Gata3 [39]. The treatment of developing Th2 cells with SH-2251 failed to inhibit the Gata3 protein expression. Therefore, it is unlikely that SH-2251 inhibits IL-5-producing Th2 cell differentiation by suppressing Ras-ERK MAPK cascade activation.

Gfi1 is a DNA binding transcriptional repressor that interacts with a number of histone modification enzymes, including LSD-1/CoRest [22], G9a [23] and HDACs [21]. However, these histone modification enzymes introduce repressive marks on the histones. We previously demonstrated that Gfi1 is required for induction of active histone marks on the *Il5* gene locus [25]. In addition, the transduction of Gfi1 into SH-2251-treated Th2 cells restored active histone modifications (H3K4me3, H3K9ac and H3K27ac) at the *Il5* gene locus. Although precious molecular mechanisms remain to be elucidated, our data indicate the possible role of Gfi1 in the formation of the active chromatin status.

An increased activity of histone acetyltransferases (HATs) and concomitant reductions in histone deacetylase (HDAC) activity have been reported in asthmatic patients [40] [41]. Changes in these histone modification enzymes result in hyperacetylations of histone, opening up the chromatin structure and increasing recruitment of RNA polymerase II [42]. Although the gene locus specific inhibitor for histone acetylation is expected to appear, such molecules have not yet been identified. We demonstrated that SH-2251 selectively inhibits induction of active histone marks, in particular H3K27ac at the *Il5* gene locus and the *Gfi1* gene locus. The transduction of *Gfi1* into SH-2251 treated Th2 cells restores the IL-5 production and active histone modifications at the *Il5* gene locus. These results indicate that SH-2251 belongs to a novel class of inhibitors that modulate histone modification status in a gene locus-specific manner.

In summary, SH-2251 selectively inhibits chromatin remodeling at the *Il5* gene locus and subsequent generation of IL-5-producing Th2 cells via attenuation of the Gfi1 expression. In addition, the oral administration of SH-2251 showed inhibitory effects on OVA-induced airway allergic inflammation. Therefore, SH-2251 is a unique class of therapeutic candidate for allergic inflammation acting through the selective inhibition of IL-5 production.

## Materials and Methods

### SH-2251

SH-2251 (United States Patent No.: US 7632865 B2) was synthesized and provided by Ishihara Sangyo Kaisha, Ltd. The purity of the SH-2251 used in the experiments was 99.1%.

### Mice

C57BL/6 and BALB/c mice were purchased from CLEA Japan. All mice were maintained under specific pathogen-free conditions and were used at 6–10 weeks of age. All experiments using mice received approval from the Kazusa DNA Research Institute Administrative Panel for Animal Care. All animal care was conducted in accordance with the guidelines of the Kazusa DNA Research Institute.

### CD4 T cells differentiation *in vitro*

Naïve CD4 T (CD44^lo^CD62L^hi^) cells were prepared using a CD4^+^CD62L^+^ T cell isolation kit II (Miltenyi Biotec). Naïve CD4 T cells (1.5×10^6^) were stimulated with an immobilized anti-TCR-β mAb (3 µg/ml; H57-597; BioLegend) and an anti-CD28 mAb (1 µg/ml; 37.5; BioLegend) with or without SH-2251 (Ishihara Sangyo Kaisha, Ltd.) under the indicated culture conditions for two days. Next, the cells were transferred onto a new plate and cultured for an additional three days in the presence of cytokines with or without SH-2251. If not mentioned, 100 nM of SH-2251 was used in the experiments. The cytokine conditions for Th2 cell differentiation were as follows: IL-2 (2.5 ng/ml), IL-4 (10 ng/ml; PeproTech) and anti-IFN-*γ* mAb (5 µg/ml; R4-6A2; BioLegend).

### Intracellular staining of cytokines

The *in vitro* differentiated Th cells were stimulated with an immobilized anti-TCR-β mAb (3 µg/ml; H57–597; BioLegend) for six hours in the presence of monensin (1 µM), and intracellular staining was performed as previously described [25]. The following antibodies were used for intracellular staining: anti-IL-4-hycoerythrin (PE) mAb (11B11; BD Bioscience), IFN-γ-FITC mAb (XMG1.2; BD Bioscience), IL-5-allophycocyanin (APC) (TRFK5; eBioscience), and IL-13-PE (eBio13A; eBioscience). A flow cytometric analysis was performed using a FACSCalibur instrument (BD biosciences), and the results were analyzed using the FlowJo software program (Tree Star).

### ELISA

The cells were stimulated with an immobilized anti-TCR-β mAb for 16 hours, and the culture supernatants were recovered. The amount of cytokines in the recovered supernatants was determined with ELISA, as described previously [43].

### Quantitative RT-PCR

Total RNA was isolated using a TRIZOL Reagent (GIBCO). cDNA was synthesized using the Superscript VILO cDNA synthesis kit (Invitrogen). Quantitative RT-PCR was performed as previously described [43], using StepOnePlus Real-Time PCR Systems (Applied Biosystems). The specific primers, and Roche Universal Probes used in the experiments were as follows:

*Hprt*: 5′ TCCTCCTCAGACCGCTTT 3′ (forward), 5′ CCTGTTCATCATCGTAATC 3′ (reverse), probe #95; *Gata3*: 5′ TTATCAAGCCCAAGCGAAG 3′ (forward), TGGTGGTGGTCTGACAGTT 3′ (reverse), probe #108; *Gfi1*: 5′ TCCGAGTTCGAGGACTTTG 3′ (forward), 5′ GAGCGGCACAGTGACTTCT 3′ (reverse), probe #7.

### Microarray analysis

The gene expression profiles of the SH-2251-treated Th2 cells were analyzed using the Agilent Whole Mouse 44K Array. The raw data were subjected to log2 transformation and normalized using the Subio Platform (Subio). The gene expression data were deposited in the GSE42131.

### Chromatin Immunoprecipitation (ChIP) assay and ChIP-sequencing

The Magna ChIP kit was used for the ChIP assay according to the manufacturer's protocol (MILLIPORE). The anti-histone H3K4me2 pAb (ab7766; Abcam), anti-histone H3K4me3 pAb (cat#39159; Activemotif), anti-histone H3K27me3 pAb (cat#39155; Activemotif), anti-histone H3K36me3 pAb (ab9050; Abcam), anti-histone H3K9ac pAb (cat#39137; ActiveMotif), anti-histone H3K27ac pAb (cat#39133; ActiveMotif), anti-Gata3 (cat# AF2605; R&D) pAb and anti-Gfi1 (M-19; Santa Cruz) were used for immunoprecipitation. The specific primers at the Th2 cytokine gene locus and the Roche Universal probes used in the experiments were as follows: #1: 5′ ACGCTTCCGGAACTAGGG 3′ (forward), 5′ CGCTCTGGCATCTCGTTC 3′ (reverse), probe #38; #2 (G2): 5′ CAGATGTGATATGCGTACATGTAATTC 3′ (forward), 5′ TGAACTCCTGACCCTGCTTT 3′ (reverse), probe #79; #3: 5′ AGTGTCTGTCCCCCAGATCA 3′ (forward), 5′ GCTGCCTGGAACTTGGTG 3′ (reverse), probe #64; #4: (Il5p), 5′ TCACTTTATCAGGAATTGAGTTTAACA 3′ (forward), 5′ GATCGGCTTTTCTTGAGCA 3′ (reverse), probe #43; #5: 5′ TGCCTCTCTTTGTTTTCCTTG 3′ (forward), 5′ GCAATTCAGTGGTAGAGT

GCTCA 3′ (reverse), probe #81; #6 (G4): 5′ AGTACAAGGGCCAAGTCACG 3′ (forward), 5′ GCCAGAGACTGGGGGTAAGT 3′ (reverse), probe #16; #7: 5′ GCTGGCCTTGAACTTACTACG 3′ (forward), 5′ GTGTGTACCCGTAATCCCA

AC 3′ (reverse), probe #10; G1: 5′ GGAAGTGGGAGTCCTAAGCA 3′ (forward), 5′ CTCCCTGCCCAACTTCTAAA 3′ (reverse), probe #15; G3: 5′ AAGGGGAGAACTGCCTCCTA 3′ (forward), 5′ TCATGCCATGGGATACAGG (reverse), prove #99; Il4p: 5′ TTGGTCTGATTTCACAGGAAAA 3′ (forward), 5′ GGCCAATCAGCACCTCTCT 3′ (reverse), probe #2; V_A_ site in the IL-4 enhancer: 5′ GCCTGTTTCCTCTCAGCATT 3′ (forward), 5′ TGATAAAAGTGACTTGAAGGTT

GG 3′ (reverse), probe #4; IL-4 intronic enhancer: 5′ CCCAAAGGAGGTGCTTTT

ATC 3′ (forward), 5′ AAATCCGAAACTGAGGAGTGC 3′ (reverse), probe #75; Il13p: 5′ CCAGGTTCTGGGTGGTTTATT 3′ (forward), 5′ GAATTACTGGGGCGGAAGTT 3′ (reverse), probe #105; Rad50p: 5′ GGAAGTGGGAGTCCTAAGCA 3′ (forward), 5′ CTCCCTGCCCAACTTCTAAA 3′ (reverse), probe #15. The specific primers at the Gfi1 gene locus and the Roche Universal probes used in the experiments were as follows:

a: 5′ TTTGCAGAAGAGTGAGGTTTGA 3′ (forward), 5′ TGGAGGCGTGGGATTAAC 3′ (reverse), probe #55; b: 5′ GACCAAGGCGTGTGA

CTATACA 3′ (forward), 5′ CACACCCTGTTGTACCCACTT 3′ (reverse), probe #48; c: 5′ GTGCCACACCACTATTCCAG 3′ (forward), 5′ AGTGGCAAAGGACCAAC

ACT 3′ (reverse), probe #2; d: 5′ TGGGGACAGGTTTTACCACT 3′ (forward), 5′ GACAGGTGGCACGAATCC 3′ (reverse), probe #70.

The samples for the ChIP-sequencing were prepared according to the manufacturer's protocol (Illumina), and the ChIP-sequence was performed using Genome Analyzer IIx (Illumina).

### Immunoblot analysis

Cytoplasmic and nuclear extracts were prepared using NE-PER Nuclear and Cytoplasmic Extraction Regents (Thermo Fisher Scientific) as previously described [43]. Anti-Gata3 mAb (HG3-31; Santa Cruz), anti-Gfi1 pAb (M-19; Santa Cruz) and anti-α-Tubulin mAb (DM1A; Lab Vision) were used for the immunoblot analysis.

### Retrovirus-mediated gene transfer

The methods for generating retrovirus supernatant and infection were described previously [25]. Infected cells were detected using staining with anti-human NGFR-PE mAb (ME20.4-1.H4; Miltenyi Biotec) and anti-PE microbeads (#130-048-801; Miltenyi Biotec), and hNGFR-positive infected cells were purified using AutoMACS (Miltenyi Biotec).

### Luciferase assay

The IL-5 promoter activity was determined as previously described [30]. In brief, M12 cells (B cell line) were cotransfected with a firefly luciferase reporter (pGL3-*Il5* promoter), a renila luciferase plasmid (pRL-TK; Promega) and an expression vector (pFlag-CMV2; Sigma) using Gene Pulser MXcell (BIO-RAD). Twenty-four hours after transfection, the cells were maintained in the presence or absence of SH-2251 for one hour, and then stimulated with PMA plus dibuteryl-cAMP for 12 hours. The luciferase activity was measured using a Dual-Luciferase Reporter Assay System (Promega).

### OVA-induced allergic airway inflammation

BALB/c mice were immunized intraperitoneally with 100 µg OVA in 2 mg of aluminum hydroxide gel on day 0. Next, the mice were intranasally challenged with OVA in saline (100 µg/mouse) on days 8 and 10. SH-2251 (10 mg/kg) was orally administered every day from day 0 to day 11. Two days after the last OVA challenge, BAL fluid cells and lung samples were prepared for histological examination as previously described [44]. Lung mononuclear cells were also prepared two days after the last OVA challenge, as previously described [45]. CD4 T cells were purified from lung mononuclear cells using anti-mouse CD4 microbeads (Miltenyi Biotec).

### Statistical analysis

Student's *t*-test was used for the statistical analyses. ANOVA and the Bonferroni-test were used in the *in vivo* experiments.

## Supporting Information

File S1 **The effects of SH-2251 on Th1-, Th9-, and Th17-differentiation.** Naïve CD4 T cells were cultured under Th1- (A), Th9- (B) or Th17- (C) conditions in the presence or absence of SH-2251 (100 nM) for five days. The cells were restimulated with an immobilized anti-TCR-β mAb for six hours, and the intracellular staining profiles were determined using intracellular staining (left). The following antibodies were used for intracellular staining: anti-IL-4-PE mAb (11B11; BD Bioscience), IFN-γ-FITC mAb (XMG1.2; BD Bioscience), anti-IL-9-PE mAb (RM9A4; BioLegend), anti-IL-17A-Alexa647 mAb (TC11-18H10.1; BioLegend) and IL-17F-Alexa488 mAb (9D3.1C8; BioLegend). The percentages of each quadrant are indicated. The cytokine production by the SH-2251-treated Th cells stimulated with an immobilized anti-TCR-β mAb for 16 hours was determined with ELISA. The culture conditions for each Th cell differentiations were as follows. Th1-conditions: IL-2 (2.5 ng/ml), IL-12 (1 ng/ml; PeproTech) and anti-IL-4 mAb (5 µg/ml; 11B11; BioLegend). Th9-conditions: IL-2 (2.5 ng/ml), IL-4 (10 ng/ml), TGF-β (10 ng/ml; PeproTech) and anti-IFN-γ mAb (5 µg/ml). The Th17-conditions were as follows: IL-6 (10 ng/ml; PeproTech), IL-1β (5 ng/ml; PeproTech), TGF-β (1 ng/ml), anti-IL-2 (5 µg/ml; BioLegend), anti-IL-4 mAb (5 µg/ml) and anti-IFN-γ mAb. Three independent experiments were performed with similar results. *P<0.05 and **P<0.01 (Student's *t*-test).
(DOCX)
