# Supplementary material for: A Novel Small Compound SH-2251 Suppresses Th2 Cell-Dependent Airway Inflammation through Selective Modulation of Chromatin Status at the Il5 Gene Locus
Source: PLoS One. 2013 Apr 16;8(4):e61785. doi: 10.1371/journal.pone.0061785 (PMC3628909; doi:10.1371/journal.pone.0061785)
Supplement: File S1 — The effects of SH-2251 on Th1-, Th9-, and Th17-differentiation. Naïve CD4 T cells were cultured under Th1- (A), Th9- (B) or Th17- (C) conditions in the presence or absence of SH-2251 (100 nM) for five days. The cells were restimulated with an immobilized anti-TCR-β mAb for six hours, and the intracellular staining profiles were determined using intracellular staining (left). The following antibodies were used for intracellular staining: anti-IL-4-PE mAb (11B11; BD Bioscience), IFN-γ-FITC mAb (XMG1.2; BD Bioscience), anti-IL-9-PE mAb (RM9A4; BioLegend), anti-IL-17A-Alexa647 mAb (TC11-18H10.1; BioLegend) and IL-17F-Alexa488 mAb (9D3.1C8; BioLegend). The percentages of each quadrant are indicated. The cytokine production by the SH-2251-treated Th cells stimulated with an immobilized anti-TCR-β mAb for 16 hours was determined with ELISA. The culture conditions for each Th cell differentiations were as follows. Th1-conditions: IL-2 (2.5 ng/ml), IL-12 (1 ng/ml; PeproTech) and anti-IL-4 mAb (5 µg/ml; 11B11; BioLegend). Th9-conditions: IL-2 (2.5 ng/ml), IL-4 (10 ng/ml), TGF-β (10 ng/ml; PeproTech) and anti-IFN-γ mAb (5 µg/ml). The Th17-conditions were as follows: IL-6 (10 ng/ml; PeproTech), IL-1β (5 ng/ml; PeproTech), TGF-β (1 ng/ml), anti-IL-2 (5 µg/ml; BioLegend), anti-IL-4 mAb (5 µg/ml) and anti-IFN-γ mAb. Three independent experiments were performed with similar results. *P<0.05 and **P<0.01 (Student's t-test). (DOCX) [file pone.0061785.s001.docx]

**Figure S1**


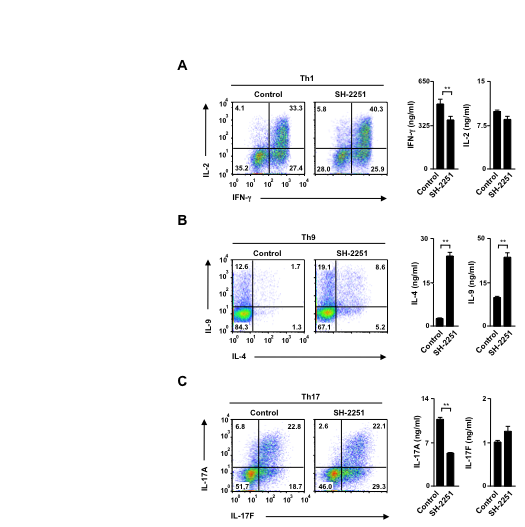


**The effects of SH-2251 on Th1-, Th9-, and Th17-differentiation.** Naïve CD4 T cells were cultured under Th1- (A), Th9- (B) or Th17- (C) conditions in the presence or absence of SH-2251 (100 nM) for five days. The cells were restimulated with an immobilized anti-TCR-β mAb for six hours, and the intracellular staining profiles were determined using intracellular staining (left). The following antibodies were used for intracellular staining: anti-IL-4-PE mAb (11B11; BD Bioscience), IFN-γ-FITC mAb (XMG1.2; BD Bioscience), anti-IL-9-PE mAb (RM9A4; BioLegend), anti-IL-17A-Alexa647 mAb (TC11-18H10.1; BioLegend) and IL-17F-Alexa488 mAb (9D3.1C8; BioLegend). The percentages of each quadrant are indicated. The cytokine production by the SH-2251-treated Th cells stimulated with an immobilized anti-TCR-β mAb for 16 hours was determined with ELISA. The culture conditions for each Th cell differentiations were as follows. Th1-conditions: IL-2 (2.5 ng/ml), IL-12 (1 ng/ml; PeproTech) and anti-IL-4 mAb (5 μg/ml; 11B11; BioLegend). Th9-conditions: IL-2 (2.5 ng/ml), IL-4 (10 ng/ml), TGF-β (10 ng/ml; PeproTech) and anti-IFN-γ mAb (5 μg/ml). The Th17-conditions were as follows: IL-6 (10 ng/ml; PeproTech), IL-1β (5 ng/ml; PeproTech), TGF-β (1 ng/ml), anti-IL-2 (5 μg/ml; BioLegend), anti-IL-4 mAb (5 μg/ml) and anti-IFN-γ mAb. Three independent experiments were performed with similar results. **P<*0.05 and ***P<*0.01 (Student’s *t*-test).

**Figure S2**

**
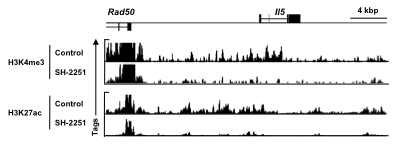
**

**The levels of H3K4me3 and H3K27ac around the *Il5* gene locus in the SH-2251-treated Th2 cells.** The histone H3K4me3 and H3K27ac patterns between the *Rad50* and *Il5* gene loci were determined using the ChIP-sequence, respectively.

**Figure S3**

**
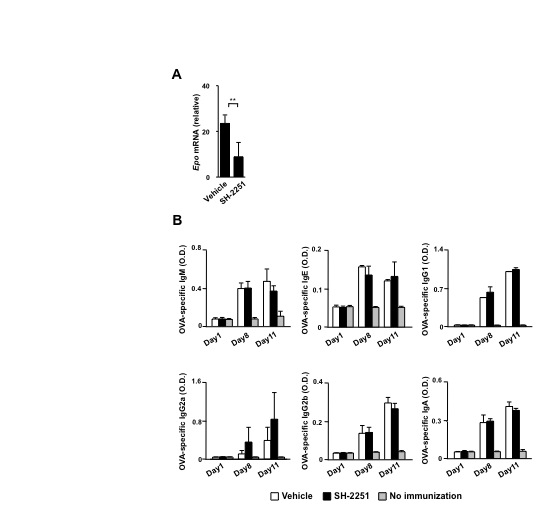
**

**Effects of SH-2251 on OVA-induced airway inflammation.**  **(A)**, The mRNA expression of *eosinophil* *peroxidase* (*Epo*) in the BAL fluid cells obtained from SH-2251-treated mice was determined with quantitative RT-PCR. **(B),** The concentrations of anti-OVA specific immunoglobulins in sera were determined with ELISA. Three independent experiments were performed with similar results. **P<*0.05 and ***P<*0.01 (Student’s *t*-test).

**Figure S4**

**
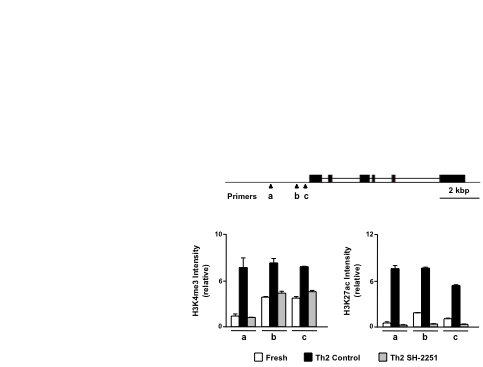
**

**The histone H3K4me3 (left) and H3K27ac (right) status at the *Gfi1* gene locus in naïve CD4 T cells, Th2 cells and SH-2251-treated Th2 cells.** A schematic representation of the *Gfi1* gene locus, and the locations of the primer pairs are also indicated (upper panel).

**Figure S5**


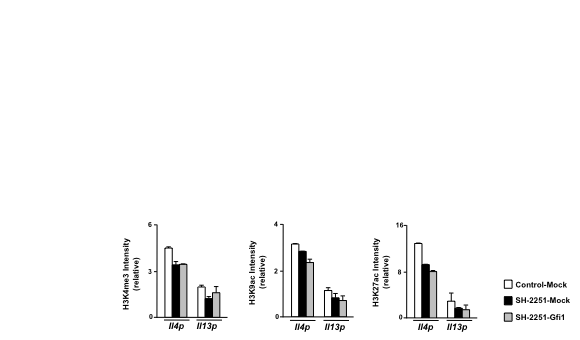


**The levels of histones H3K4me3 and H3K27ac at the *Il4* and *Il13* promoters were not influenced by transduction of *Gfi1*.** The levels of histones H3K4me3, H3K9ac and H3K27ac at the *Il4* and *Il13* gene loci in the hNGFR-positive *Gfi1*-transduced SH-2251-treated Th2 cells. The relative intensity (/Input) is shown with the standard deviation.
